# Multidrug-resistant isolates from Ukrainian patients in a German health facility: a genomic surveillance study focusing on antimicrobial resistance and bacterial relatedness

**DOI:** 10.1007/s15010-023-02061-4

**Published:** 2023-06-20

**Authors:** Claudia Stein, Maria Zechel, Riccardo Spott, Mathias W. Pletz, Frank Kipp

**Affiliations:** Institute for Infectious Diseases and Infection Control, Jena University Hospital, Am Klinikum 1, 07747 Jena, Germany

**Keywords:** Antimicrobial resistance, Carbapenemases, Infection control, cgMLST, Incidence rate, Community-acquired

## Abstract

**Purpose:**

Antimicrobial resistance is a pressing issue in Ukraine, with healthcare-associated infections caused by multidrug-resistant organisms being a major concern. A recent prospective multicenter study revealed a staggering rate of 48.4% antimicrobial resistance to carbapenems among Enterobacterales causing a healthcare-associated infection. We conducted a systematic survey to investigate the incidence rate and incidence density of carbapenemase-producing Gram-negative bacteria (CPGN) among refugees and war-wounded Ukrainians in connection with the German health system.

**Methods:**

From the onset of the war until November 2022, seven Ukrainian patients were admitted to our hospital. Upon admission, screening samples and samples from the focus of suspected infection were taken from all seven patients. The incidence rate and the incidence density of CPGN were calculated as a result of the microbiological findings. We sequenced all CPGN using Illumina technology.

**Results:**

The incidence rate of CPGN at our hospital was 0.06 for 2021 and 0.18 for 2022. All seven Ukrainian patients were infected or colonized with at least one CPGN, including *K. pneumoniae* (14/25)*, P. aeruginosa* (6/25), *A. baumannii* (1/25), *Providencia stutartii* (1/25), *C. freundii* (1/25), and *E. coli* (2/25)*.* Genomic surveillance revealed that (i) most frequently detected carbapenemases among all sequenced isolates were *bla*_NDM_ (17/25) and *bla*_OXA-48_ (6/25), (ii) most commonly observed plasmid replicons among the *K. pneumoniae* isolates recovered from Ukrainian patients were Col(pHAD28) (12/14), IncHI1B(pNDM-MAR) (9/14), IncFIB(pNDM-Mar) (12/14), and (iii) clonal relation between the pathogens of the Ukrainian isolates, but not for the isolates from our hospital surveillance system.

**Conclusion:**

The rising prevalence of community-acquired colonization and infection with CPGN is having a direct effect on the infection prevention measures, such as higher number of isolations, reprocessing of patient rooms, additional microbiological testing and overall organization within hospitals.

**Supplementary Information:**

The online version contains supplementary material available at 10.1007/s15010-023-02061-4.

## Introduction

### Antimicrobial resistance: political and epidemiological factors get together

Antimicrobial resistance has changed the ways in which therapeutic approaches to bacterial infections and other ailments must be approached. Responsible and judicious use of antibiotics, increased research into developing new and better treatments, and proper education are all essential to mitigating the potentially dangerous consequences of antimicrobial resistance [1]. Antimicrobial resistance is a pressing issue in Ukraine, with healthcare-associated infections caused by multidrug-resistant organisms being a major concern [2, 3]. A recent prospective multicenter study revealed a staggering rate of 48.4% antimicrobial resistance to carbapenems among Enterobacterales causing a healthcare-associated infection [4]. The European Centre for Disease Prevention and Control (ECDC) has warned that the ongoing war in Ukraine could lead to the spread of antimicrobial resistance throughout Europe [5]. Despite this, only a few scientific reports have been published on the matter [6–8]. The genomic surveillance of multidrug-resistant organisms from Ukrainian patients in the Netherlands and Germany revealed globally spread epidemic lineages and high number of NDM-1 beta-lactamase. The shortcomings of national surveillance reports are their lack of denominator data. Without denominator data, it is difficult to accurately assess the incidence rate of multidrug-resistant organisms.

We conducted a systematic survey to investigate the incidence of multidrug-resistant Gram-negative bacteria among refugees and war-wounded Ukrainians in connection with the German health system, based on whole-genome sequencing analysis. This study aimed to shed light on the incidence of these bacteria among Ukrainians.

## Methods

### Recruitment

At our University Hospital, we have 1411 beds across 26 clinics. Every year, we provide care for around 53,600 inpatients and carry out over 274,000 outpatient consultations. As the only university hospital and facility for basic, standard, and maximum health care in the region of Thuringia (a federal state in Germany), our hospital serves over one million citizens. This study encompasses all clinics and institutes of our hospital. From the onset of the war until November 2022, seven Ukrainian patients were admitted to our hospital. Three of these seven patients were hospitalized due to war wounds, while the remaining four were refugees. On-site surgery was performed to treat the wounded soldiers. Upon admission to our clinic, screening samples and samples from the focus of suspected infection were taken from all seven patients. Patient samples were analyzed in an accredited medical microbiology laboratory using standard operating procedures. The incidence rate and incidence density of CPGN were calculated based on the microbiological findings. The incidence rate was calculated for 2021 and 2022 and given in relation to 100 patient cases. The incidence density was also calculated for 2021 and 2022 and given in relation to 1000 patient days.

We sequence all CPGN at our hospital, regardless of whether colonization or infection had occurred. This enabled us to create a database of over a thousand whole-genome sequenced pathogens.

### Genomic characterization of multi-drug-resistant isolates via whole-genome sequencing

For microbiological diagnostics, the sample was streaked onto Columbia sheep blood agar (BD, Heidelberg, Germany) and Drigalski lactose agar (Oxoid, ThermoFisher Scientific, Wesel, Germany) for overnight incubation at 37 °C. Colonies were identified by Vitek MS (bioMérieux, Nürtingen, Germany). Antimicrobial susceptibility testing (AST) was performed by determination of minimal inhibitory concentrations (MIC) using Vitek 2 (bioMérieux) and the MICRONAUT-S MDR MRGN Screening microtiter system (Merlin Diagnostika, Bornheim, Germany; distributed by Sifin Diagnostics, Berlin, Germany). Twenty-five isolates from seven Ukrainian patients were sequenced using an Illumina MiniSeq instrument (Illumina, San Diego, CA, USA). DNA was extracted directly from plated microbial cultures using the NexteraTM DNA Flex Microbial Colony Extraction protocol (Illumina, San Diego, CA, USA). The fastq file format was then assembled using Velvet and analyzed with the Ridom SeqSphere + software version 8.4.1. To analyze the species-specific clonal relationship, core genome multilocus sequence typing (cgMLST) was employed. Resistance determinants and plasmids were screened by querying genome assemblies against the ResFinder and PlasmidFinder databases (PlasmidFinder version 2.1.6 (2021-03-27); ResFinder version: 2022-03-17) using the tool ABRicate with minimal coverage and minimal identity settings of 80%, respectively [9–11]. The results were then compared to our existing database to assess clonal relatedness and antimicrobial resistance.

## Results

### War-wounded and refugees within the German health-care system

**Fig. 1:**
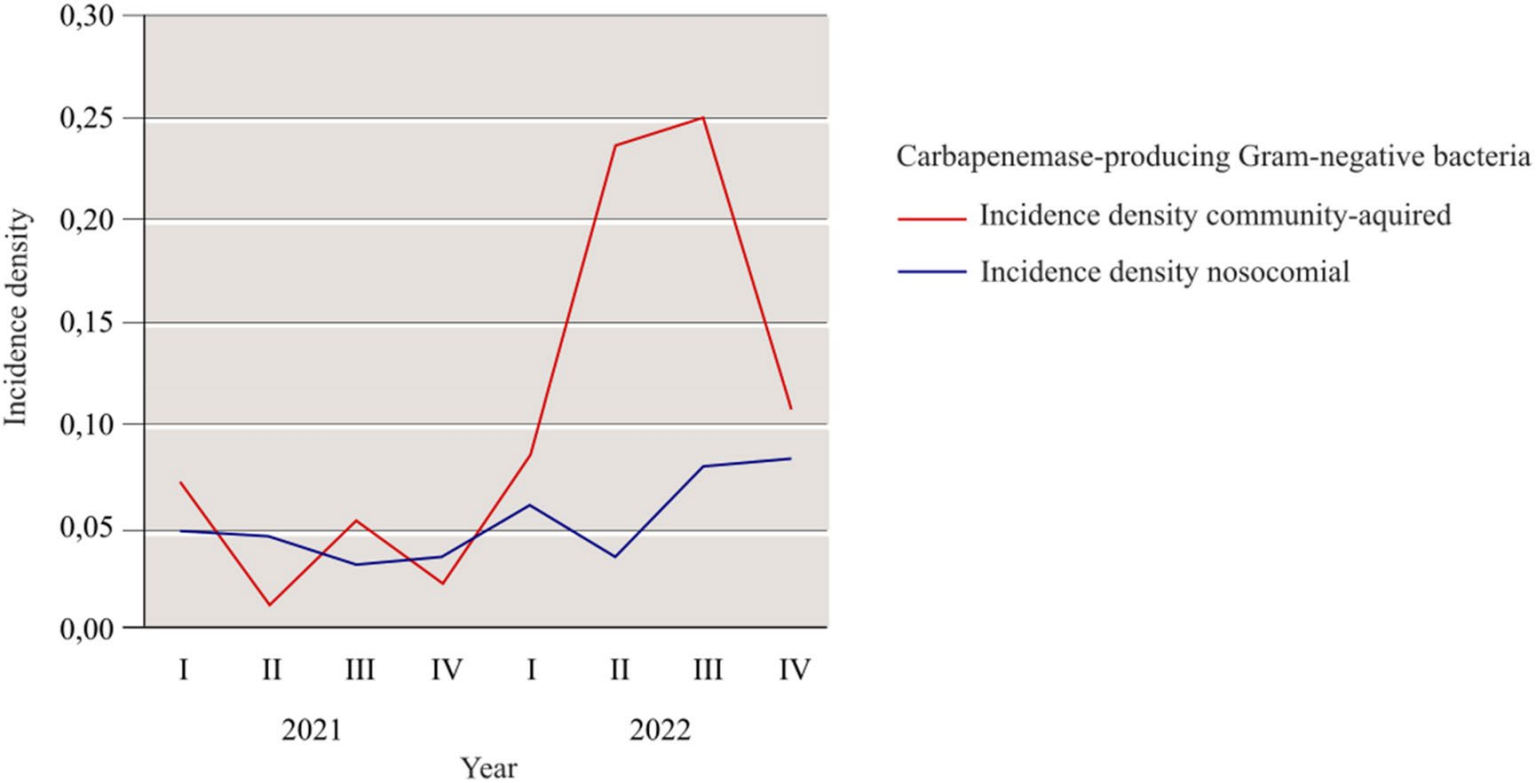
The incidence density of CPGN at our hospital for 2021 and 2022. The values were divided into community-acquired and nosocomial cases

The incidence rate of CPGN at our hospital rose from 0.06 in 2021 to 0.18 in 2022. There was also an increase in incidence density from 2021 (0.08) to 2022 (0.24). This increase was partially attributed to an outbreak of CPGN, which was not associated with Ukrainian patients, resulting in a slight increase in nosocomial cases. However, a significant surge in community-acquired cases was observed in the second and third quarters of 2022 (see Fig. 1), mainly due to the high number of detected CPGN among Ukrainian patients during this period.

All seven Ukrainian patients were infected or colonized with at least one CPGN, including carbapenemase-producing *P. aeruginosa*, *A. baumannii*, *K. pneumoniae*, and *Providencia stutartii*, *C. freundii*, and *E. coli* (see Table 1). The minimum inhibitory concentration values for the antibiotics that were tested have been provided in supplementary information (SI 1). Three wounded soldiers, who had already been treated in Ukraine, were found to have wound infections caused by CPGN, as well as colonization with other carbapenemase-producing species. All patients with an infection received a consultation by an Infectious Diseases physician and were treated with antibiotics tailored to the pathogen detection (e.g., cefiderocol).

**Table 1:** Clinical data of the seven Ukrainian patients were collected. Patients #1–#4 were admitted as refugees, while patients #5–#7 were wounded soldiers

Patient-identifier	Admission date	Age, sex	Cause of admission	Sampling	Infection/colonization	Isolated pathogen	Carbapenemase
#1	March	76 years, female	Transfer from external hospital with known myeloproliferative disease	Stool	Colonization	*K. pneumoniae*	NDM-3
#2	April	54 years, female	Dialysis patient with acute hepatitis b	Urine	Colonization	*P. aeruginosa*	NDM-1
#3	August	3 years, male	Suspected obstructive sleep apnea syndrome with known mucopolysaccharidosis	Rectal swab Rectal swab	Colonization Colonization	*K. pneumoniae* *K. pneumoniae*	OXA-48 NDM-1
#4	September	49 years, female	Transfer from external hospital with liver cirrhosis	Urine Blood culture Rectal swab Ascites	Colonization Infection Colonization Infection	*K. pneumoniae* *K. pneumoniae* *K. pneumoniae* *K. pneumoniae*	NDM-1 NDM-1 NDM-1 NDM
#5	April	48 years, male	Direct transfer from Ukraine due to war injuries Wounded 04/2022 Multiple bony injuries of the lower extremities on both sides Urologic and abdominal injury from a bullet through the abdomen	Wound Blood culture Abdomen Rectal swab Rectal swab Urine	Infection Infection Infection Colonization Colonization Colonization	*P. aeruginosa* *K. pneumoniae* *A. baumannii* *E. coli* *K. pneumoniae* *K. pneumoniae*	IMP-34 NDM-1 OXA-72, -90 NDM-5 OXA-48 NDM-5, OXA-48
#6	June	47 years, male	Direct transfer from Ukraine due to war injuries Wounded 05/2022 Open fractures of the upper and lower extremities	Skin Skin	Colonization Colonization	*C. freundii* *P. aeruginosa*	NDM-1 IMP-34
#7	August	35 years, male	Direct transfer from Ukraine due to war injuries Wounded 05/2022 Mine injury with fractures of the lower extremities	Blood culture Catheter Catheter Rectal swab Rectal swab Rectal swab Deep wound Deep wound Deep wound	Infection Colonization Colonization Colonization Colonization Colonization Infection Infection Infection	*K. pneumoniae* *K. pneumoniae* *P. aeruginosa* *K. pneumoniae* *K. pneumoniae* *E. coli* *P. aeruginosa* *P. aeruginosa* *P. stutartii*	OXA-48 NDM-1 NDM-1 NDM-1, OXA-48 NDM-1, OXA-48 KPC-3 NDM-1 VIM-2 NDM

### High prevalence of antimicrobial resistance genes and mobile genetic elements

**Fig. 2:**
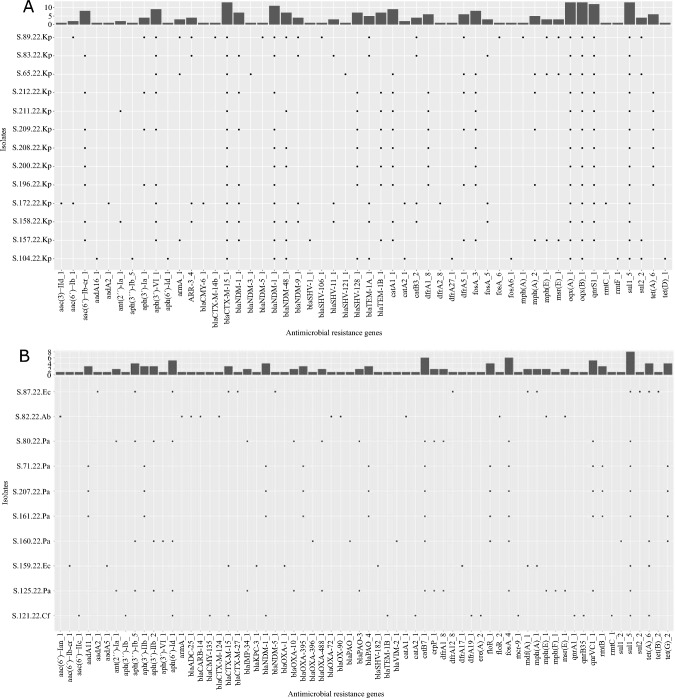
Illustration of the abundance and diversity of antimicrobial resistance genes within the *K. pneumoniae* (Kp) in part **A** and *P. aeruginosa* (Pa), *A. baumannii* (Ab), *C. freundii* (Cf) and *E. coli* (Ec) species in part **B**

The most frequently detected carbapenemases among all sequenced isolates were *bla*_NDM_ (17/25) and *bla*_OXA-48_ (6/25). The ESBL enzyme CTX-M-15 (16/25) and beta-lactamase SHV-182 (8/25) were predominantly found in *K. pneumoniae* (see Fig. 2A), *C. freundii*, and *E. coli*. Notably, the prevalence of these enzymes varied significantly between the species.

Most *P. aeruginosa* isolates were found to carry *bla*_PAO_ genes (6/6) as well as *bla*_OXA_ variants such as *bla*_OXA-395_ (3/6), *bla*_OXA-396_ (1/6) and *bla*_OXA-488_ (2/6) (see Fig. 2B). Notably, one *C. freundii* isolate was found to contain the newly identified *mcr-9* resistance gene [12], which confers resistance to colistin, an antimicrobial drug of last resort used to treat serious Gram-negative bacterial infections. Additionally, several genes encoding resistance to aminoglycosides (*aac(6´)-Ib-cr*, *aph(3´)-VI*), chloramphenicol (*catA1*, *catA2*), fosfomycin (*fosA*), fluoroquinolones (*oqxA*, *oqxB*, *qnrS*), sulfonamides (*sul1*, *sul2*), and tetracyclines (*tet*(A)) were observed in the majority of the different species. The resistance genes identified in the Ukrainian isolates are well known and can be found also in our database. There, carbapenemase-forming bacteria were more prevalent in patients with a history of prolonged hospitalization or travel. It is important to highlight that the frequency of detecting resistance genes, particularly carbapenemases, in Ukrainian patients' isolates significantly differs from those in our database. Furthermore, the presence of plasmid replicons was also examined.

**Fig. 3:**
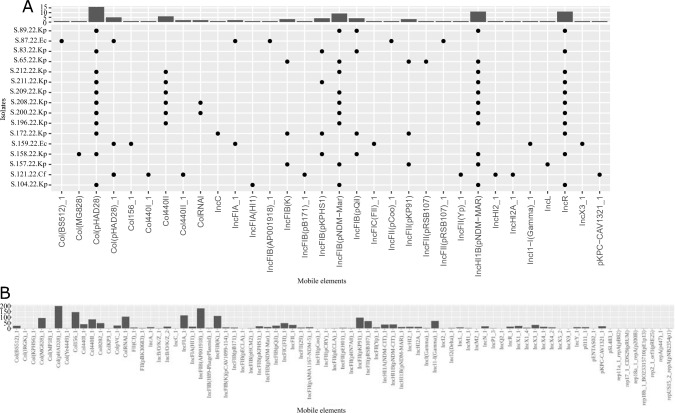
Mobile genetic element distribution of the Ukrainian isolates (part **A**). *C. freundii* isolates were abbreviated within the isolate name with .Cf, *K. pneumoniae* isolates with .Kp and *E. coli* with .Ec. **B** Mobile genetic element distribution of isolates collected in our hospital previously (part **B**)

The most commonly observed plasmid replicons among the *K. pneumoniae* isolates recovered from Ukrainian patients were Col(pHAD28) (12/14), IncHI1B(pNDM-MAR) (9/14), and IncFIB(pNDM-Mar) (12/14). Plasmid pNDM-MAR, a member of the IncH plasmid family, is a well-known bla_NDM-1_-bearing plasmid. In a few of the clinical isolates from our surveillance database, we also detected the IncHI1B(pNDM-MAR) and IncFIB(pNDM-Mar) replicons from *K. pneumoniae* isolates. The Col(pHAD28) replicon was the most frequently observed and is representative of carbapenemase-producing isolates from our *K. pneumoniae* database. Plasmid replicons from the same family were also detected in *E. coli* and *C. freundii* isolates from Ukrainian patients (see Fig. 3). Furthermore, we have identified the plasmid replicons also in our surveillance database, as illustrated in Fig. 3B.

Comparative analysis of the antimicrobial resistance genes and the plasmid replicons revealed no within-patient plasmid transfer between the different Gram-negative species, causing colonization or infection of the patients.

We investigated the epidemiological connection between Ukrainian isolates and the clinical isolates in our database. We observed a clonal relationship between the pathogens for the Ukrainian isolates, but not for the isolates from our clinical surveillance database. No nosocomial transmissions were detected within our hospital. However, due to the limited medical history and language barrier, it is not possible to evaluate whether there is a possible connection within the home country. For *K. pneumoniae*, we mostly detected isolates belonging to sequence type 395 (7/13) among the Ukrainian isolates. In our surveillance database, we have identified several MLST sequence types (STs) of bacterial isolates. Among these, ST307 (7% of all *K. pneumoniae* isolates in our database), ST147 (6%), and ST45 (6%) are the most prevalent in *K. pneumoniae*. Additionally, we have observed ST395 (6%) to be the most frequently detected ST in our database for this species.

For *E. coli*, we have sequenced MLST ST131 (*E. coli* MLST Warwick, 29%) most frequently in our database. Conversely, ST167 and ST46 of the Ukrainian isolates are only sporadically present or not at all in our database. In *P. aeruginosa*, we have identified MLST ST235 (31%) as the most commonly detected ST in our database. The STs isolated from Ukrainian patients (ST654, ST1047, ST773) have yet to appear in our database. Notably, we isolated pathogens from the same species without high genomic similarity and different antimicrobial resistance profiles within one focus, e.g., wounds (see Table 1, patients #3, #5, #7). This phenomenon is not usually observed for our daily, routinely infectious cases, but from the wounded soldiers.

## Discussion

### Principal findings

We determined an increase in the incidence rate and incidence density for carbapenemase-producing bacteria from 2021 to 2022 at our hospital. This rise is mainly due to the high number of carbapenem-resistant isolates that we detected in Ukrainian patients. All Ukraine patients, who were admitted at our hospital until November 2022 were colonized or infected with at least one CPGN, which are insensitive to reserve antibiotics. High prevalence of these pathogens causes extensive infection prevention measures. In addition, the risk of nosocomial transmission increases and poses the danger of life-threatening infections that can no longer be treated with antibiotics. Furthermore infections are a major contributor to the rising cost of healthcare [13]. A case–control study revealed that patients who tested positive for carbapenem-resistant *Enterobacteriaceae* had an elevated risk of having received certain antimicrobials and undergoing invasive procedures. This heightened risk highlights the need for increased education on antimicrobial stewardship and infection control measures to prevent the emergence and spread of carbapenemase-producing bacteria in healthcare settings [14]. Especially the war-wounded patients revealed at least two carbapenemase-forming pathogens per patient, harboring different carbapenemases. This reflects a cross-infection of the wounded patients contaminated with exogenous environmental bacteria and nosocomially transmitted pathogens from first aid on-site as one potential mode of infection and colonization [15]. Alternative explanations may include an origin in the patient’s endogenous colonization flora or transmission events during evacuation transports. A German case report also corresponds to the complexity of caring for war-wounded and other patients from Ukraine and the risk of spreading antimicrobial resistance, but without sequencing results [7].

The increased incidence rate for carbapenemase-producing bacteria within our hospital point up the importance of stringent screening protocols and pre-emptive isolation measures in clinical settings, particularly for war-wounded patients. This proactive approach is critical to ensure the safety of the patients most at risk. Another way to protect vulnerable patient groups from colonization and infection with carbapenemase-producing bacteria is to utilize biocides such as chlorhexidine for bathing patients [16]. However, we have already identified chlorhexidine-resistant *K. pneumoniae* isolates, raising doubts about the efficacy of this approach [17].

Our report substantiates the recommendations of the ECDC [4], and confirms previously published surveillance data from Germany and the Netherlands, regarding the identified sequence types and resistance genes [6, 8]. In these investigations, *bla*NDM and *bla*OXA-48 were also the most commonly detected beta-lactamase. Furthermore, we confirmed the high prevalence for the sequence type ST395 for *K. pneumoniae*. Regarding the distribution of plasmids, the detected replicons are well-known and have been isolated from patients in West Europe [18]. What is worrying is the prevalence of such plasmids, carrying genes encoding metallo-, extended-spectrum β-lactamase, and a range of resistance genes against other antibiotic groups, even with the small number of cases within our report.

We were able to calculate the incidence rate of carbapenem-resistant strains due to our systematic survey of all patients in our hospital. However, this study is limited to the patients of one hospital. One limitation of the present survey is the small number of patients we could include in our observations. Furthermore, long-read sequencing is the more suitable method to characterize plasmid replicons. For economic reasons, the isolates were not additionally sequenced using a long-read technique.

The incidence rate and incidence density of CPGN have been on the rise, resulting in a greater number of patients being isolated. This has had a significant impact on the organization of examinations and inpatient processes within hospitals, leading to an increase in the number of comprehensive final disinfections. Our clinic has been successful in preventing nosocomial transmission, which validates the efficacy of the hygiene measures and barriers we have implemented. However, the increasing incidence rate of carbapenemase-producing pathogens also highlights the importance of active screening and pre-emptive isolation.

## Conclusion

The increasing prevalence of community-acquired colonization and infection with CPGN is increasing epidemiologic pressure on inpatient care. As a result, drastic and costly infection prevention measures are needed to avoid nosocomial transmission and life-threatening infections.

Seamless microbiological surveillance and whole-genome sequencing can create an effective early warning system to combat this issue.

### Supplementary Information

Below is the link to the electronic supplementary material.

Supplementary file1 (DOCX 19 KB)

## Data Availability

Whole genome sequencing (WGS) data are available under study ID PRJEB58365 (ERP143427) at the European Nucleotide Archive (ENA).

## Abbreviations

ST: Sequence type
Cf: *C. freundii*
Kp: *K. pneumoniae*
Ec: *E. coli*
cgMLST: Core genome multilocus sequence typing
CPGN: Carbapenemase-producing Gram-negative bacteria
MIC: Minimal inhibitory conentration
ECDC: European Centre for Disease Prevention and Control
