# Supplementary material for: Multidrug-resistant isolates from Ukrainian patients in a German health facility: a genomic surveillance study focusing on antimicrobial resistance and bacterial relatedness
Source: Infection. 2023 Jun 20;51(6):1731–8. doi: 10.1007/s15010-023-02061-4 (PMC10665453; doi:10.1007/s15010-023-02061-4)
Supplement: Supplementary file 1 — Supplementary file1 (DOCX 19 KB) [file 15010_2023_2061_MOESM1_ESM.docx]

**Supplementary information 1** Microbial characterization (Minimal inhibitory concentrations in mg/l) of the isolates collected from seven Ukrainian patients. U: unassigned sequence type.

| Isolate | MLST sequence type | Tigecyclin | Aztreonam-avibactam | Amoxicillin | Ampicillin-sulbactam | Fosfomycin | Piperacillin-tazobactam | Cefotaxim | Ceftazidim | Ceftazidim-avibactam | Ceftotaxim-tazbactam | Cefiderocol | Meropenem | Ciprofloxacin | Cotrimoxazol | Gentamicin | Colistin | Nitrofurantoin | Amikacin | Levofloxacin | Tobramycin | Aztreonam | Amoxicillin-clavulanic acid | Moxifloxacin |
| --- | --- | --- | --- | --- | --- | --- | --- | --- | --- | --- | --- | --- | --- | --- | --- | --- | --- | --- | --- | --- | --- | --- | --- | --- |
| S.196.22.Kp | 395 | 0.5 | 1/4 |  |  |  |  |  |  |  |  |  |  |  |  |  |  |  |  |  |  |  |  |  |
| S.209.22.Kp | 395 | 0.5 | 1/4 |  |  |  |  |  |  |  |  |  |  |  |  |  |  |  |  |  |  |  |  |  |
| S.212.22.Kp | 395 | 0.5 | 1/4 |  |  |  |  |  |  |  |  |  |  |  |  |  |  |  |  |  |  |  |  |  |
| S.211.22.Kp | 395 |  | 1/4 | ≥32 | ≥32 | ≥256 | ≥128 | ≥64 | ≥64 | 1/4 | 32/4 | 0.38 | ≥16 | ≥4 | ≥32 | ≥16 | 0.5 |  | 4 | >2 |  |  |  |  |
| S.172.22.Kp |  |  |  | ≥32 |  | 64 | ≥128 | ≥64 | ≥64 | >16/4 | >16/4 | 0.38 | >128 | ≥4 | ≥3 | ≥32 | ≤1 |  | >32 | >2 |  |  |  |  |
| S.157.22.Kp | 395 | ≤0.25 |  | ≥32 | ≥32 | ≥256 | ≥128 | ≥64 | ≥64 | >16/4 | >8/4 | 8 | ≥16 | ≥4 | ≥32 | ≥16 | ≤1 |  | >32 | >2 |  |  |  |  |
| S.158.22.Kp | 147 | ≤0.25 |  | ≥32 | ≥32 | ≥256 | ≥128 | ≥64 | ≥64 | >16/4 | >8/4 | 1 | ≥16 | ≥4 | ≥32 | ≥16 | ≤1 |  | 8 | >2 |  |  |  |  |
| S.159.22.Ec | U | 0.5 |  | ≥32 | ≥32 | ≤16 | ≥128 | ≥64 | ≥64 | 8/4 | 8/4 | 12 | 8 | ≥4 | ≥32 | ≤1 | ≤1 | 64 | ≤4 | >2 |  |  |  |  |
| S.160.22.Pa | 654 |  |  |  |  | 128 | ≥128 |  | ≥64 | 8/4 | 8/4 | 0.125 | ≥16 | ≥4 |  |  | ≤1 |  | 8 | >8 | ≥16 | 16 |  |  |
| S.161.22.Pa | 773 |  |  |  |  | 128 | ≥128 |  | ≥64 | 8/4 | 8/4 | 1 | ≥16 | ≥4 |  | ≥32 | ≤1 |  | >32 | >8 | ≥16 | 4 |  |  |
| S.200.22.Kp | 395 |  | 1/4 |  |  | 128 |  |  | >32 | 1/4 | 32/4 | 0.023 | ≥16 | >2 |  |  | 0.5 |  | 8 |  |  |  |  |  |
| S.207.22.Pa | U |  |  | ≥32 |  | ≥256 | ≥128 | ≥64 | ≥64 | 1/4 | 8/4 | 0.25 | ≥16 | ≥4 | ≥32 |  | ≤1 |  | 8 | >2 |  |  | ≥64 |  |
| S.208.22.Kp | 395 |  |  |  |  |  | ≥128 |  | ≥64 | 8/4 | 8/4 | 0.19 | ≥16 | ≥8 |  |  | ≤1 |  | 32 |  | ≥16 | ≥64 |  |  |
| S.121.22.Cf | U |  |  | ≥32 | ≥32 | ≤32 | ≥128 | ≥64 | ≥64 |  |  | 0.38 | ≥16 | ≥4 | ≥32 |  |  |  |  |  |  |  |  | ≥8 |
| S.125.22.Pa | 1047 |  |  |  |  |  | ≥128 |  | ≥64 | 8/4 | 8/4 | 0.19 | ≥16 | ≥8 |  |  | ≤1 |  | 32 |  | ≥8 | ≥64 |  |  |
| S.126.22.Ab | U |  |  |  |  | >128 |  |  |  | >8/4 |  | 0.125 | 4 | ≥4 | ≥32 | ≤1 | ≤1 |  |  |  |  |  |  |  |
| S.80.22.Pa | 1047 |  |  |  |  | 64 | ≥128 |  | ≥64 |  |  |  | ≥16 | ≥4 |  |  | 1 |  | ≥64 |  | ≥16 | ≥64 |  |  |
| S.82.22.Ab | U | 0.25 |  |  |  |  |  |  |  |  |  | 0.38 | 16 | ≥4 | ≥32 | ≥16 | 1 |  |  |  |  |  |  |  |
| S.83.22.Kp | U | <0.25 |  |  |  | 4 | >64/4 | >2 | >128 | >16/4 | >8/4 | 0.094 | 32 | >2 |  |  | <1 |  | 8 | >2 |  |  |  |  |
| S.87.22.Ec | U |  |  |  |  | <32 | >64/4 |  | >128 | >16/4 | >8/4 |  | 16 | >2 |  |  | ≤1 |  | ≤4 | >2 |  |  |  |  |
| S.89.22.Kp | U |  |  | ≥32 |  | ≤16 |  |  | >128 | ≥64 | ≥64 |  | 1 | ≥4 | ≥32 |  |  |  |  | ≥8 |  |  | ≥32 |  |
| S.104.22.Kp | 11 |  |  |  |  |  | >128 |  | ≥64 |  |  | 0.38 | ≥16 | ≥4 |  |  | <1 |  |  | ≥8 |  | 8 |  |  |
| S.71.22.Pa | 773 | 0.5 |  | 2 |  |  | ≥128 |  | ≥64 | >16/4 | >8/4 | 0.38 | ≥16 | ≥4 | ≥32 | ≤1 | ≤1 |  | >32 |  |  |  |  |  |
| S.65.22.Kp | 874 | 4 |  | ≥32 | ≥32 |  | ≥128 | ≥64 | ≥64 |  |  | 1 | ≥16 | ≥4 | ≥32 | ≥16 |  |  |  |  |  |  |  | ≥8 |
